# Long‐Term Clinical Outcomes of Combined Polydioxanone and Thin Nonabsorbable Polypropylene Thread Lifting in Korean Adults Aged 60 Years and Older

**DOI:** 10.1111/jocd.71216

**Published:** 2026-11-02

**Authors:** Young Keun Lee, Sung Hun Jung, Jin‐Su Chung, Guk Hyun Shin, Soo Yeon Park, Kyu‐Ho Yi

**Affiliations:** ^1^ The Beauty Plastic Surgery Clinic Seoul Republic of Korea; ^2^ Made Young Plastic Surgery Clinic Seoul Korea; ^3^ Division in Anatomy and Developmental Biology, Department of Oral Biology Yonsei University College of Dentistry Seoul South Korea; ^4^ You and I Clinic Seoul Republic of Korea

**Keywords:** aged, bruising, combined thread lift, dimpling, facial rejuvenation, Korean patients, long‐term follow‐up, nonabsorbable thread, polydioxanone, polypropylene

## Abstract

**Background:**

In adults aged 60 years and older with advanced facial laxity or ptosis, surgical facelift remains the gold‐standard and most predictable treatment. Thread lifting should therefore be considered a minimally invasive option for appropriately selected patients rather than an equivalent substitute for rhytidectomy. This study evaluated the long‐term effectiveness and safety of a combined absorbable polydioxanone (PDO) and thin nonabsorbable polypropylene (PP) thread‐lifting strategy in an older Korean cohort.

**Methods:**

This retrospective, consecutive, single‐center case series included 243 Korean patients treated from January 2018 through December 2020. Mean age was 64.3 years (range, 60–78 years), and 221 patients (90.9%) were women. Patients had moderate‐to‐severe facial ptosis (Grade 2–3 using the clinic's routine grading system). Treatment used a standardized bilateral construct of 36 threads (Long V threads), each 20 cm long, delivered through nine entry points per hemiface: nine thicker PDO threads and nine thinner PP threads per side. The PDO component was intended to provide greater initial mechanical tissue engagement, whereas the permanent PP component was intended to provide ongoing structural support over time. Median follow‐up was 24 months (range, 6–36 months). Effectiveness was assessed using serial clinical photographs and nonblinded physician‐assessed Global Aesthetic Improvement Scale (GAIS) scores; safety events were abstracted from clinical records.

**Results:**

Follow‐up was available for 243/243 patients at 6 months, 218/243 (89.7%) at 1 year, 167/243 (68.7%) at 2 years, and 89/243 (36.6%) at 3 years. Mean GAIS scores were 4.2 ± 0.6, 3.9 ± 0.7, and 3.5 ± 0.8 at 6 months, 1 year, and 2 years, respectively. Bruising occurred in 78 patients (32.1%) and resolved within 2 weeks; edema occurred in 45 (18.5%), asymmetry in 12 (4.9%), infection in 1 (0.4%), and granuloma in 1 (0.4%). Two patients (0.8%) required hook‐assisted thread removal for persistent dimpling. Selected longitudinal photographs showed qualitative persistence of contour change for up to 3 years, but photography was nonstandardized and was not used as an objective quantitative endpoint.

**Conclusions:**

Combined PDO‐PP thread lifting was associated with persistent clinical improvement in selected Korean adults aged 60 years and older. However, the noncomparative design, nonstandardized photography, nonblinded GAIS assessment, follow‐up attrition, and absence of pure‐PDO or pure‐PP comparator groups prevent attribution of early or late effects to either material and preclude claims of superiority or synergy. In patients with advanced skin excess or cervicofacial ptosis, surgical facelift remains the reference treatment; thread lifting should be reserved for carefully selected patients after realistic counseling regarding magnitude, durability, complications, and the potential need for removal of a malpositioned permanent thread.

## 1 Introduction

Minimally invasive facial rejuvenation has expanded from skin‐directed procedures to interventions intended to reposition the mobile soft‐tissue envelope. Thread lifting occupies an intermediate position between nonlifting treatments and open surgical rhytidectomy. Barbed or cogged threads engage soft tissue along a selected vector and may produce immediate mechanical repositioning, followed over time by tissue integration around the implanted material [1, 2, 3, 4, 5]. In patients with substantial skin excess, advanced jowling, or cervicofacial ptosis, however, surgical facelift remains the gold‐standard and most predictable treatment; thread lifting should be framed as a less invasive option for appropriately selected patients rather than as an equivalent alternative [4, 5, 6].

The clinical challenge is particularly pronounced in adults aged 60 years or older. Facial aging is a composite process involving reduced dermal elasticity, redistribution and deflation of superficial and deep fat compartments, attenuation or displacement of retaining structures, altered muscular balance, and skeletal remodeling. Older patients may therefore present with a heavier and less elastic soft‐tissue envelope, deep nasolabial and marionette folds, jowling, and loss of mandibular definition. A thread that produces a visible change in a younger patient with mild laxity may be insufficient in an older patient with substantial tissue mass and advanced ptosis. Conversely, increasing local thread density may increase procedural burden, bruising, tissue bunching, dimpling, palpability, and dissatisfaction. The central clinical question is therefore not how many threads can be inserted, but whether an adequately distributed and anatomically safe construct can achieve a realistic degree of correction in a properly selected patient.

Absorbable threads made from polydioxanone (PDO), polycaprolactone (PCL), or poly‐L‐lactic acid‐containing materials are widely used because the polymer gradually resorbs and long‐term exposure to a permanent implant is avoided. Clinical series have reported satisfactory short‐term and, in some studies, 1 to 2‐year outcomes with PDO and other absorbable systems [7, 8, 9, 10, 11, 12, 13, 14]. Immediate improvement after placement is primarily mechanical, reflecting engagement and repositioning of tissue; collagen deposition and biological remodeling develop later and should not be described as the cause of the immediate lift [15, 16, 17]. As tensile integrity diminishes with absorption, the remaining biological response may not reproduce the mechanical fixation of an intact permanent suture, and recurrence or maintenance treatment may occur.

Nonabsorbable polypropylene offers a different risk–benefit profile. Because the polymer remains in tissue, it can continue to function as a structural element after early edema and tissue rearrangement have resolved. Park et al. reported high satisfaction with a fine medical‐grade polypropylene thread in 102 patients, whereas Fukaya described longer‐term behavior after insoluble thread lifting [18, 19]. Other permanent‐thread series, however, have reported subtle or short‐lived improvement, revision, palpability, and removal [3, 4]. Thus, the permanence of the material does not itself establish long‐term stability. Durability depends on thread design, fixation, insertion plane, vector, tissue characteristics, patient selection, and technique, while the persistent implant introduces specific considerations regarding reversibility, foreign‐body reaction, infection, extrusion, visibility, palpability, asymmetry, and the possibility of later removal.

Safety considerations are especially important in older patients. A systematic review and meta‐analysis found that older age and nonabsorbable materials were associated with higher risks of selected complications, including dimpling, infection, paresthesia, and extrusion [20]. More recent large analyses of PDO procedures likewise identified older age and higher thread count as predictors of complications or dissatisfaction [21, 22]. These findings do not preclude thread lifting in older adults, but they reinforce the need for conservative patient selection, distributed rather than excessive thread density, careful plane control, and explicit counseling about both temporary and permanent‐material complications.

The present study describes a large retrospective clinical experience with a standardized combined thread‐lifting construct in 243 Korean adults aged 60 years or older. The protocol paired thicker absorbable PDO threads, selected for greater initial mechanical engagement, with thin nonabsorbable PP threads intended to provide continuing structural support. The objectives were to describe the persistence of clinical improvement, summarize recorded safety outcomes, present selected longitudinal cases as qualitative illustrations, and critically define the clinical role and limitations of this temporary–permanent construct. Because every patient received both materials, the study cannot isolate the contribution of either component, establish synergy, or demonstrate superiority over single‐material treatment.

## 2 Materials and Methods

### 2.1 Study Design and Setting

This was a retrospective, consecutive, single‐center case series of treatments performed from January 2018 through December 2020 at The Beauty Plastic Surgery Clinic, Seoul, Korea. The cohort comprised 243 unique Korean patients who contributed 243 index treatment episodes. Follow‐up ranged from 6 to 36 months. Consecutive sampling was used to reduce discretionary case selection. The study was designed to characterize routine‐practice effectiveness and safety of a standardized combined PDO‐PP protocol in older patients rather than to test a prespecified superiority hypothesis.

### 2.2 Ethical Considerations

The study was conducted in accordance with the Declaration of Helsinki. Consent for treatment and retrospective analysis was obtained.

### 2.3 Patient Selection

Eligible patients were Korean adults aged 60 years or older with moderate‐to‐severe age‐related facial ptosis classified as Grade 2–3 using the clinic's routine facial‐ptosis grading system, who underwent the combined PDO‐PP protocol and had at least 6 months of clinical follow‐up. Treatment was directed to one or more of the midface, nasolabial region, marionette region, jowl, and mandibular contour. Baseline abstraction included age, sex, ptosis severity, hypertension, diabetes, and prior facial procedures. Patients taking anticoagulant or antiplatelet drugs and those with active infection or autoimmune disease were excluded.

### 2.4 Thread Materials and Construct

The standardized construct combined two LongV lifting‐thread types. The permanent polypropylene (PP) component was a thin cog thread (USP 2–0) paired with a 20‐G, 120‐mm cannula. The absorbable PDO component was thicker (USP No. 1) and paired with an 18‐G, 120‐mm cannula. Each PP and PDO thread measured 20 cm (200 mm). The thicker PDO threads were selected for greater initial mechanical engagement of the mobile soft tissue, whereas the thinner permanent PP threads were intended to provide continuing structural support after the absorbable component lost tensile integrity. This assignment describes the procedural rationale only; the study did not include component‐specific comparator groups and therefore cannot establish the independent effect of either material. Clinical behavior also depends on barb geometry, directionality, elasticity, fixation, and insertion plane [18, 23, 24, 25, 26].

### 2.5 Preprocedural Assessment and Vector Planning

Treatment planning was individualized according to the dominant direction of descent, tissue weight, skin mobility, and available anchoring regions. Figure 1 shows the nine entry points used on one hemiface and the distributed PDO and PP trajectories extending from the lateral temporal‐zygomatic region toward the mid and lower face; the same configuration was used contralaterally. The purpose of multiple distributed vectors was to share load over a broad area rather than concentrate traction at a single point. In advanced ptosis, a single lateral vector may not adequately address the nasolabial, marionette, jowl, and mandibular regions, and additional vectors may be required to support different mobile compartments [23, 24, 27]. Vector planning was performed with the patient upright, with attention to avoiding lower‐eyelid distortion, exaggerated malar fullness, or an unnatural lateral sweep.

**FIGURE 1:**
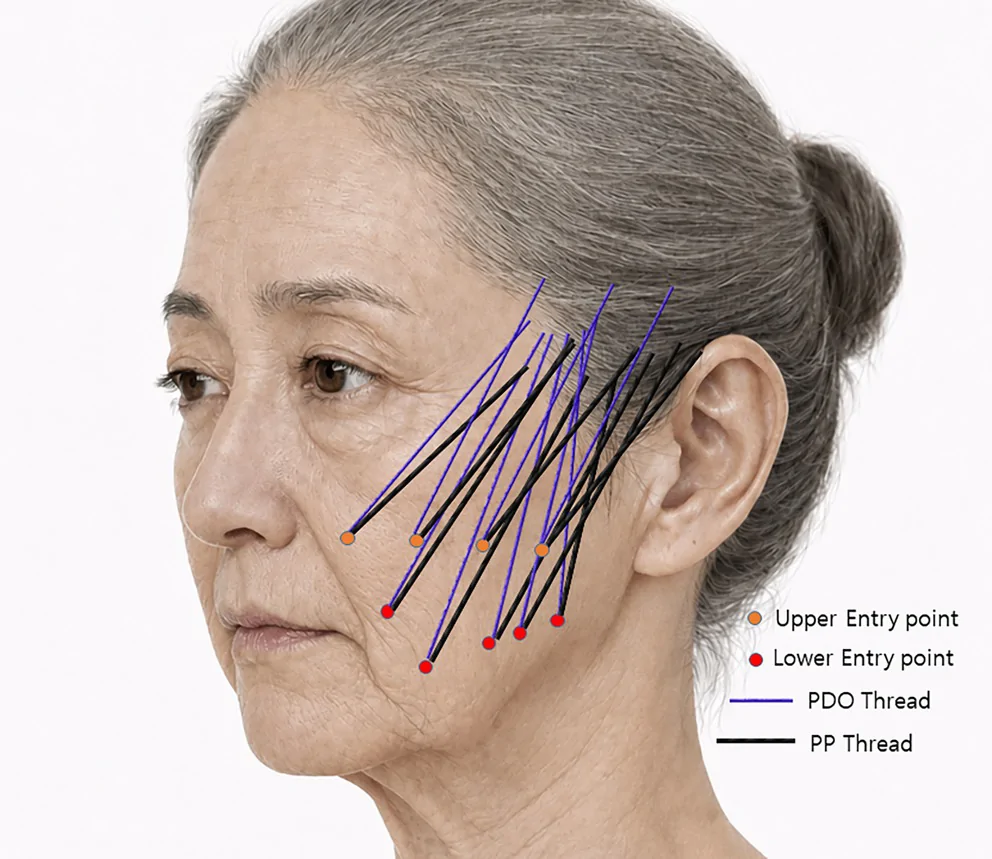
Representative combined PDO‐PP vector plan shown on one hemiface. Nine entry points were used to distribute nine thicker PDO threads and nine thinner PP threads per side. The same configuration was repeated contralaterally, yielding 36 threads in total. PDO was selected for greater initial mechanical tissue engagement, whereas PP was intended to provide continuing support; the component‐specific effects were not independently tested.

### 2.6 Procedural Technique

Tumescent solution was infiltrated throughout the treatment area, and the designated thread entry points were positioned above the nasolabial fold and marionette region. After skin preparation and sterile draping, nine entry points and the planned trajectories were marked on each side with the patient upright. No sedation was administered, and postoperative antibiotics were prescribed. The intended insertion plane was subcutaneous or deep dermal according to the treated region. Superficial placement can cause linear visibility, tethering, and persistent dimpling, whereas excessively deep passage may reduce the intended skin‐envelope effect and may place the parotid gland, parotid duct, facial nerve branches, or vessels at risk [28, 29, 30, 31] (Figure 1).

A total of 36 threads were placed during each treatment episode. Nine PP and nine PDO threads were distributed through nine entry points on each side, resulting in 18 threads per hemiface and 36 threads overall. Every thread was 20 cm long. The thicker PDO threads were intended to create greater initial mechanical tissue engagement, while the thinner nonabsorbable PP threads were intended to provide continuing support. Because all patients received both components, the protocol cannot quantify the independent contribution of PDO or PP and should not be interpreted as evidence of biological or mechanical synergy.

### 2.7 Postprocedural Care and Follow‐Up

Patients were followed clinically for 6 to 36 months, with assessment points at 6 months, 1 year, 2 years, and 3 years. Serial clinical photography was available at these intervals, and physician‐assessed GAIS scores were available through 2 years. The retrospective safety abstraction included bruising, edema, asymmetry, infection, granuloma, persistent dimpling, and thread removal. A validated patient‐reported pain score was not available, and pain was therefore not analyzed as an effectiveness or safety endpoint.

### 2.8 Outcome Definitions

Effectiveness outcomes were physician‐assessed GAIS scores at 6 months, 1 year, and 2 years and qualitative persistence of lifting or contour change on serial clinical photographs. Under the clinic's scoring convention, higher GAIS values indicated greater improvement. No cohort‐wide mean GAIS value was available at 3 years. Because the photographs were obtained during routine clinical care and were not consistently standardized for pose, lighting, camera distance, expression, hair position, or viewing angle, they were treated as qualitative adjuncts and not as objective measurements of displacement. Safety outcomes included bruising and time to resolution, edema, asymmetry, infection, granuloma, persistent dimpling, and hook‐assisted removal. An aggregate chart category recorded as “no severe problem” was not operationally defined and was therefore not treated as a validated serious‐adverse‐event endpoint.

### 2.9 Management of Persistent Dimpling

Early dimpling may reflect temporary tissue bunching and may improve as edema decreases and engaged tissues redistribute. Persistent focal depression raises concern for excessively superficial engagement, a fixed barb, focal tethering, or unfavorable vector geometry. In two patients, dimpling persisted and the involved PP thread was removed using a minimally invasive hook‐based approach. Hook extraction has been described as a method for locating and removing a superficially misplaced thread through a small access point [32].

### 2.10 Statistical Analysis

Descriptive statistics were used. Continuous outcomes are presented as means with standard deviations or ranges, and categorical outcomes as frequencies and percentages. Follow‐up and GAIS values were summarized using the available‐case denominator at each time point; no imputation was performed. No hypothesis testing, survival analysis, multivariable modeling, or direct comparison with pure PDO, pure PP, surgical facelift, or another thread construct was performed because this was a single‐arm retrospective case series without a concurrent comparator group.

## 3 Results

### 3.1 Cohort Characteristics

The cohort comprised 243 Korean patients aged 60 years or older, with a mean age of 64.3 years (range, 60–78 years). There were 221 women (90.9%) and 22 men (9.1%). Baseline facial ptosis was categorized as moderate to severe (clinic‐used Grade 2–3 classification). Hypertension was recorded in 76 patients (31.3%) and diabetes mellitus in 34 (14.0%); these comorbidities were not mutually exclusive. Forty‐five patients (18.5%) had undergone a prior facial procedure, although the type and timing were not available in the retrospective summary. Each treatment episode used the standardized 36‐thread construct: 18 threads per side, comprising 9 thicker PDO and 9 thinner PP threads delivered through nine entry points per hemiface (Table 1).

**TABLE 1:** Cohort and treatment characteristics.

Characteristic	Value
Patients	243
Age, mean (range), years	64.3 (60–78)
Women	221 (90.9%)
Men	22 (9.1%)
Facial ptosis	Moderate to severe; clinic‐used Grade 2–3 classification
Hypertension	76 (31.3%)
Diabetes mellitus	34 (14.0%)
Prior facial procedure	45 (18.5%)
Threads per treatment	36 total; 18 per hemiface
Material per hemiface	9 PDO + 9 PP threads
Thread length	20 cm each
Entry points	5 per hemiface
Median follow‐up (range)	24 months (6–36 months)

### 3.2 Long‐Term Clinical Persistence

Median follow‐up was 24 months (range, 6–36 months). All 243 patients were assessed at 6 months; 218/243 (89.7%) had 1‐year follow‐up, 167/243 (68.7%) had 2‐year follow‐up, and 89/243 (36.6%) had 3‐year follow‐up (Table 2). Selected clinical photographs showed qualitative persistence of contour change at 6 months and at 1, 2, and 3 years (Figures 2, 3, 4, 5, 6). Because image acquisition was not standardized, the photographs were not used to quantify mandibular displacement, jowl elevation, midface position, or other objective changes.

**TABLE 2:** Follow‐up availability and physician‐assessed GAIS.

Time point	Patients available, *n*/*N* (%)	Mean GAIS ± SD
6 months	243/243 (100)	4.2 ± 0.6
1 year	218/243 (89.7)	3.9 ± 0.7
2 years	167/243 (68.7)	3.5 ± 0.8
3 years	89/243 (36.6)	Not assessed cohort‐wide

**FIGURE 2:**
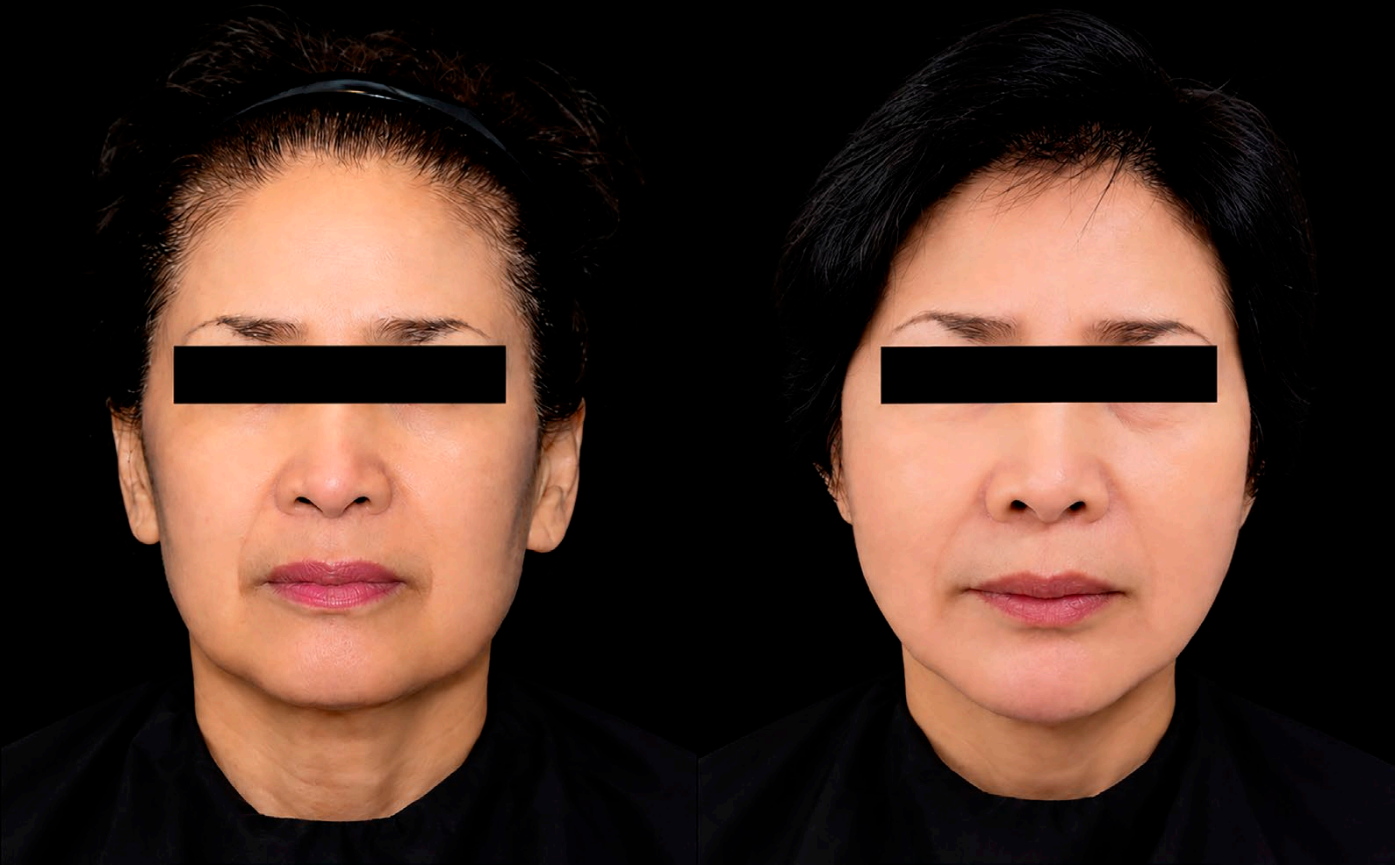
Illustrative Case 1. Baseline (left) and 1‐year follow‐up (right) after combined PDO‐PP thread lifting. A qualitative contour difference remains visible at follow‐up. Photography was not fully standardized and should not be interpreted as an objective measurement of lift.

**FIGURE 3:**
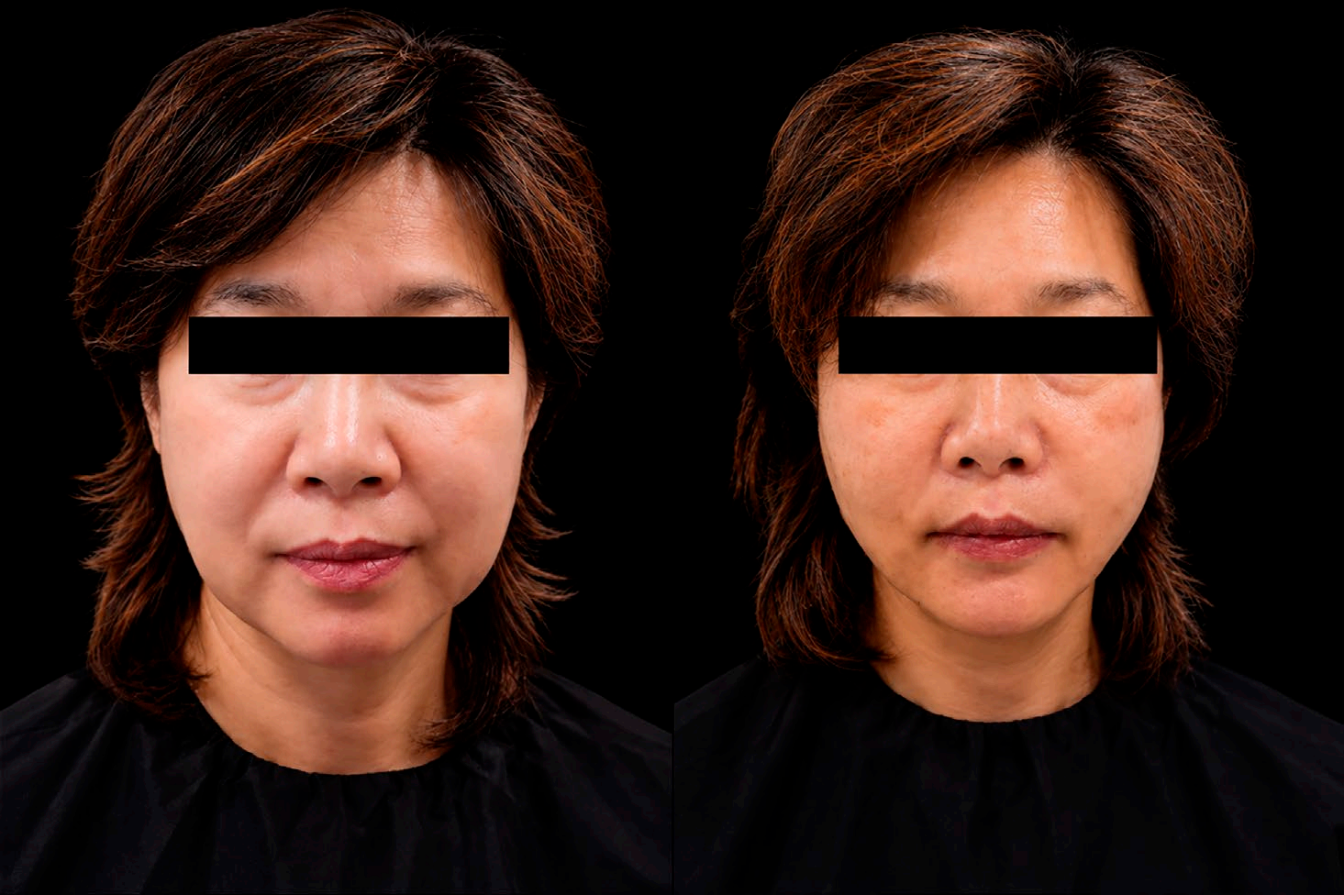
Illustrative Case 2. Baseline (left) and 2‐year follow‐up (right) after combined PDO‐PP thread lifting. The pair is presented as a qualitative longitudinal illustration; differences in lighting, camera setup, hair position, and clothing limit objective comparison.

**FIGURE 4:**
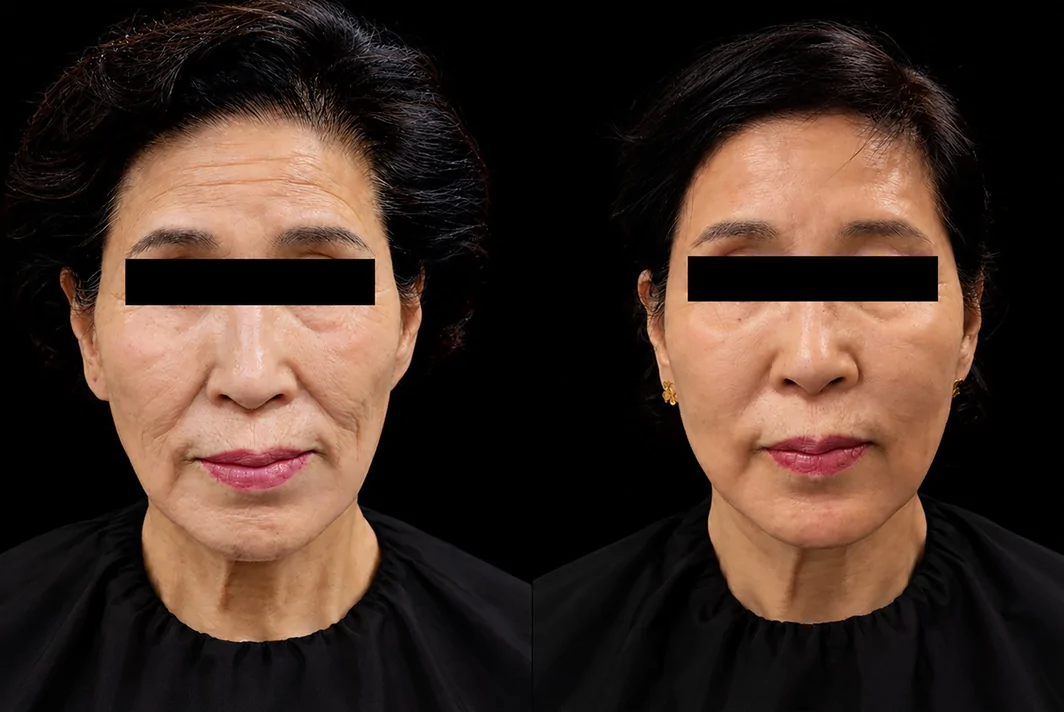
Illustrative Case 3. Baseline (left) and 3‐year follow‐up (right) after combined PDO‐PP thread lifting. Residual contour change is visible in this selected patient, but the nonstandardized image pair cannot establish quantitative durability.

**FIGURE 5:**
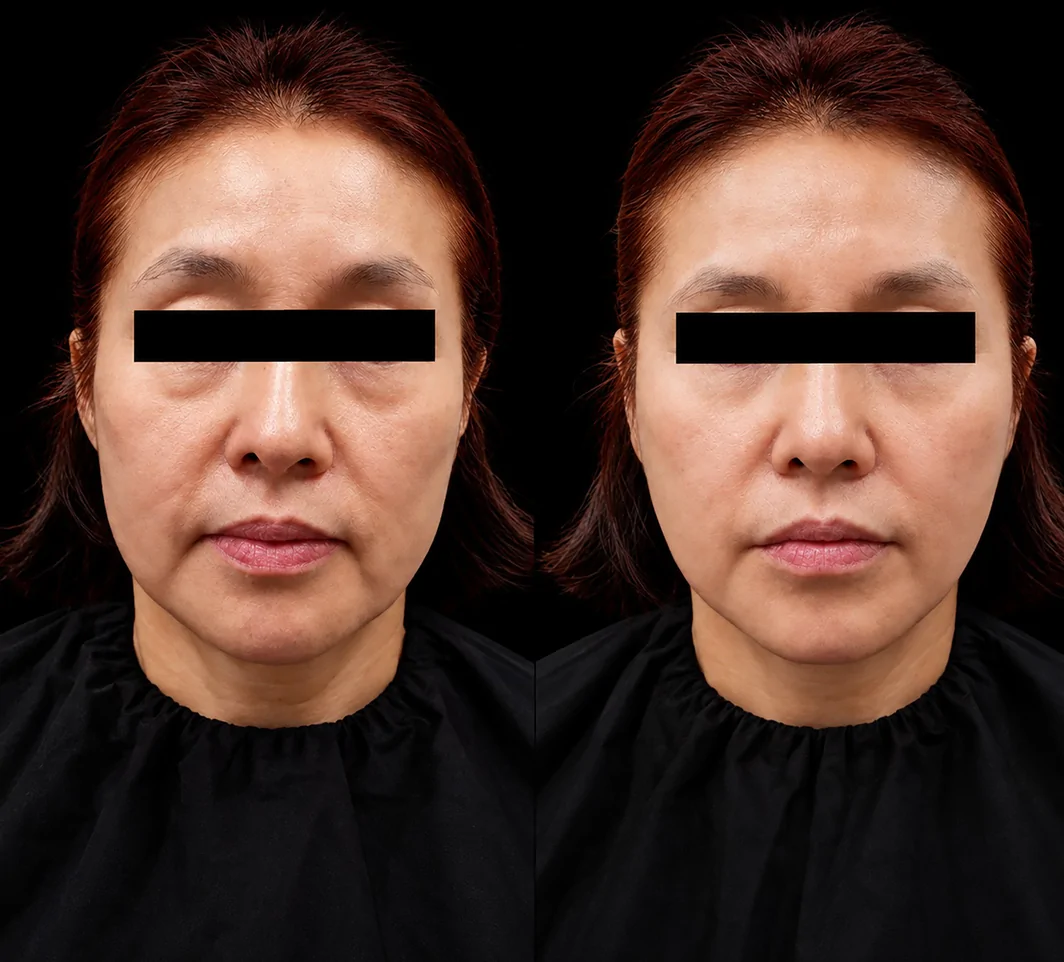
Illustrative Case 4. Baseline (left) and 3‐year follow‐up (right) after combined PDO‐PP thread lifting. The images provide qualitative long‐term context only and were not used for objective grading.

**FIGURE 6:**
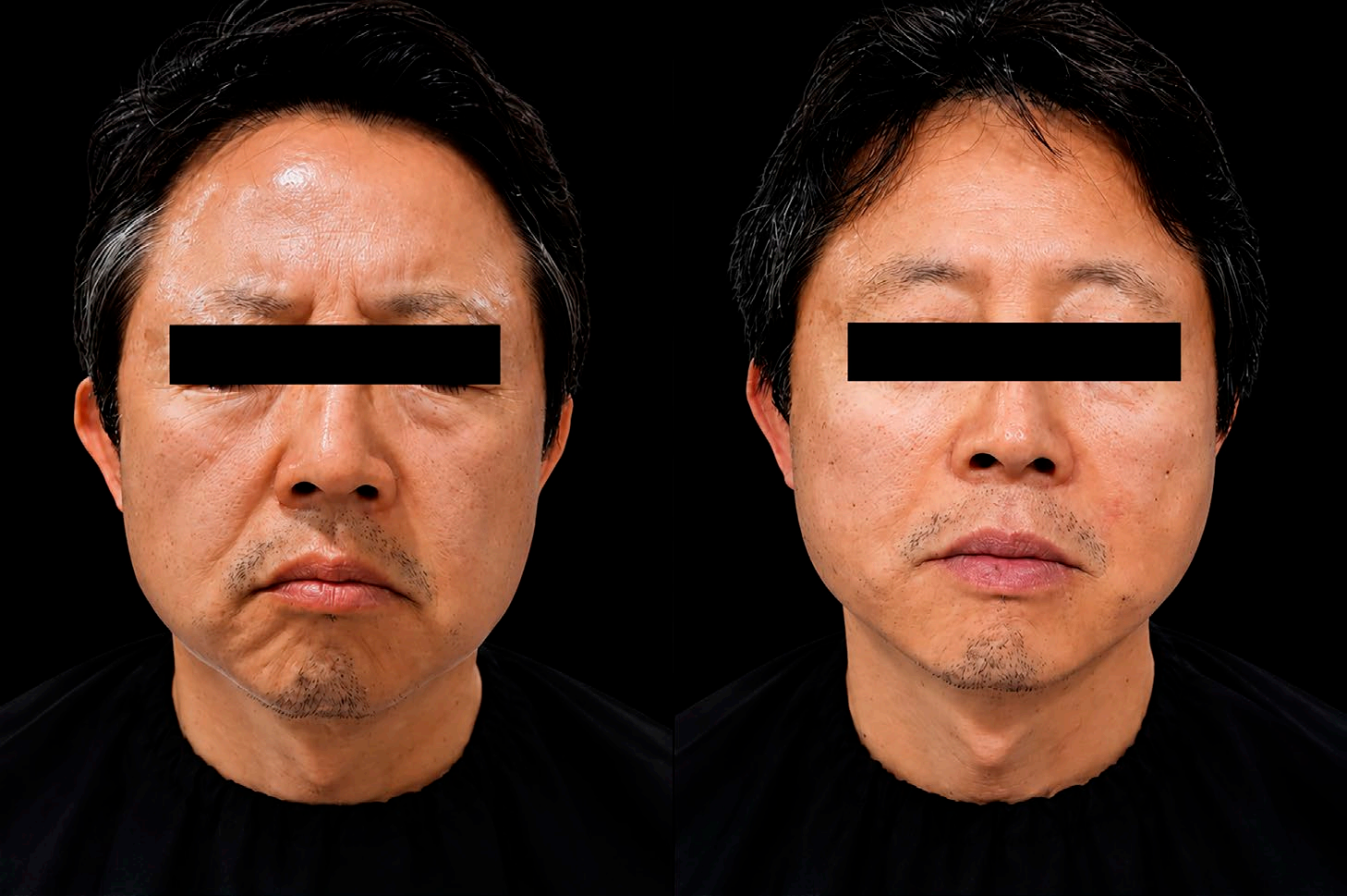
Illustrative Case 5. Baseline (left) and 6‐month follow‐up (right) after combined PDO‐PP thread lifting. Facial expression and eye position differ between images; the pair is illustrative and should not be used for objective grading.

Mean physician‐assessed GAIS scores were 4.2 ± 0.6 at 6 months (*n* = 243), 3.9 ± 0.7 at 1 year (*n* = 218), and 3.5 ± 0.8 at 2 years (*n* = 167) (Table 2). The 0.7‐point decline from 6 months to 2 years was consistent with gradual attenuation of the physician‐rated improvement. Because assessment was nonblinded and no pure‐PDO, pure‐PP, surgical, or alternative‐thread comparator was included, these values describe persistence after the combined protocol but do not establish the independent contribution of either material or superiority over another treatment.

GAIS values were physician‐assessed and nonblinded; available‐case denominators were used at each time point.

### 3.3 Safety Outcomes

An aggregate chart summary designated 238/243 patients (97.9%) as having “no severe problem”; because this category was not operationally defined, it is reported descriptively and was not used as a validated safety endpoint. Bruising occurred in 78 patients (32.1%) and resolved within 2 weeks in all recorded cases. Edema occurred in 45 (18.5%), asymmetry in 12 (4.9%), infection in 1 (0.4%), and granuloma in 1 (0.4%) (Table 3). The retrospective summary did not provide uniform onset, duration, severity grading, management, or patient‐level co‐occurrence for all adverse‐event categories.

**TABLE 3:** Recorded adverse events.

Event	*n*/*N* (%)	Recorded information
Bruising	78/243 (32.1)	Resolved within 2 weeks in all recorded cases
Edema	45/243 (18.5)	Uniform duration not available
Asymmetry	12/243 (4.9)	Uniform duration not available
Infection	1/243 (0.4)	Recorded event
Granuloma	1/243 (0.4)	Recorded event
Persistent dimpling requiring hook‐assisted removal	2/243 (0.8)	Intervention required
Chart summary: “no severe problem”	238/243 (97.9)	Descriptive only; category not operationally defined

Two patients (2/243, 0.8%) developed persistent dimpling that did not resolve with observation and required hook‐assisted removal of the involved PP thread. One infection and one granuloma were also recorded. These events are clinically relevant because a permanent implant does not resorb spontaneously and may require targeted release or removal when malposition, persistent tethering, infection, granulomatous reaction, visibility, palpability, extrusion, or other implant‐related problems occur.

### 3.4 Illustrative Longitudinal Cases

Case 1 is shown at baseline and 1 year (Figure 2), Case 2 at baseline and 2 years (Figure 3), Cases 3 and 4 at baseline and 3 years (Figures 4 and 5), and Case 5 at baseline and 6 months (Figure 6). These cases are retained solely as qualitative longitudinal illustrations. They were not used for objective grading or comparative efficacy claims because frontal, oblique, and lateral views were not consistently standardized across time points, and routine‐care differences in pose, expression, lighting, camera distance, hair position, and background may influence visual interpretation.

## 4 Discussion

### 4.1 Principal Findings

This retrospective series of 243 Korean adults aged 60 years or older describes outcomes after a standardized thread PDO‐PP construct. Three observations are clinically relevant. First, physician‐rated improvement persisted through 2 years, with mean GAIS declining from 4.2 ± 0.6 at 6 months to 3.5 ± 0.8 at 2 years, while selected patients had qualitative photographic follow‐up to 3 years. Second, bruising and edema were the most frequent recorded events, and two patients required hook‐assisted removal for persistent dimpling. Third, the study design supports only an association between the combined protocol and observed outcomes; it cannot determine whether PDO, PP, or their combination was responsible for early or late effects.

### 4.2 Interpretation of Durability and the Combined Construct

The procedural rationale for the combined construct was temporal complementarity. Nine thicker PDO threads per side were selected for greater initial mechanical engagement, while nine thinner permanent PP threads per side were intended to provide continuing structural support after the absorbable component lost tensile integrity. Immediate correction after any thread lift is primarily mechanical and may also be influenced by edema and temporary tissue compression; collagen deposition and tissue remodeling occur later. The present data therefore should not be interpreted as evidence that PDO produces an immediate biological lift or that PP independently guarantees long‐term stability.

Fukaya's long‐term insoluble‐thread study is relevant because it separated early and later behavior conceptually: the apparent rejuvenation effect decreased during the first year and later showed an increasing trend, which was interpreted as possible ongoing suspension rather than uninterrupted preservation of the immediate postoperative correction [19]. In the present cohort, the decrease in mean GAIS between 6 months and 2 years is compatible with partial attenuation of the initial correction. Selected 2 and 3‐year photographs show that visible contour differences can remain in some patients, but nonstandardized photography and 36.6% retention at 3 years prevent a quantitative claim of stable long‐term lift.

The literature on permanent threads is not uniformly favorable. Kaminer et al. reported moderate sustained improvement up to 16 months with anchored barbed sutures, [3] whereas Garvey et al. described subtle, short‐lived results, revision, and removal of palpable threads [4]. Differences in thread design, thickness, fixation, plane, vector, patient anatomy, and operator technique likely contribute to this heterogeneity. The present study should therefore be interpreted as experience with one specific combined PDO‐PP protocol rather than evidence applicable to all PP or absorbable thread systems.

### 4.3 Permanent Versus Resorbable Threads: Clinical Trade‐Offs

Absorbable PDO and permanent PP threads have distinct clinical trade‐offs. PDO provides temporary tensile support and gradually resorbs; this may be attractive when reversibility and avoidance of a permanent implant are priorities, but loss of tensile integrity can be followed by recurrence and the need for maintenance treatment. PP remains in tissue and can continue to provide mechanical support, but permanence also means that a visible, palpable, extruded, infected, granulomatous, asymmetrical, or malpositioned thread does not resolve through polymer absorption and may require release or removal. Neither material should therefore be described as categorically superior. Choice of material should reflect tissue burden, desired duration, acceptance of a permanent implant, reversibility, complication tolerance, and the operator's ability to manage removal [3, 4, 6, 18, 19, 20, 25, 30, 32].

In this protocol, the thicker PDO component was intended to improve initial mechanical engagement, while the thin PP component was intended to preserve support over a longer interval. The observed 2‐year GAIS values and selected 3‐year photographs are compatible with persistence after the combined protocol, but they do not establish that the combination lasts longer than PDO alone or PP alone. A scientifically stronger design would compare pure‐PDO, pure‐PP, and combined groups using standardized imaging, blinded assessment, patient‐reported outcomes, and identical follow‐up intervals.

### 4.4 Clinical Positioning in Adults Aged 60 Years and Older

Older faces often have less elastic recovery and more heterogeneous tissue descent. Superficial and deep fat compartments may deflate or descend, retaining structures may create abrupt transitions between fixed and mobile tissue, and skeletal support changes with age. A lateral thread vector applied to a heavy central compartment may therefore produce limited displacement, bunching near a retaining structure, or early recurrence. These anatomic features explain why treatment in older patients requires particularly careful selection and realistic counseling [23, 24, 26, 27].

The premise that older patients simply “need more threads” should be rejected. Recent studies of PDO thread lifting found that older age and higher thread count independently predicted dissatisfaction or complications [21, 22]. Thread number should therefore be interpreted together with vector distribution, tissue load, insertion plane, fixation, and the amount of correction that can realistically be achieved without excessive local density.

For advanced skin excess, marked jowling, and cervicofacial ptosis, surgical facelift remains the gold‐standard and most predictable treatment. Thread lifting should not be presented as capable of reproducing a well‐performed rhytidectomy in this setting [4, 5, 6]. A minimally invasive thread procedure may still be considered in selected older patients when the expected degree of improvement is modest and clearly discussed. The clinician should determine whether the patient's dominant problem is realistically thread‐responsive before proceeding and should avoid compensating for poor indication by simply increasing the number of threads.

### 4.5 Treatment Burden and Pain

Treatment burden is an important part of clinical value. Placement of 36 individual 20‐cm threads requires multiple long cannula passes even though puncture sites were limited to nine entry points per side. The present retrospective dataset did not include a validated pain score, analgesic consumption, or time to recovery, so pain‐related claims cannot be made. This limitation is particularly relevant in older patients for whom a technically intensive minimally invasive procedure may still produce substantial short‐term burden.

Future prospective studies should record procedural pain, postprocedural pain, analgesic use, downtime, time to return to normal mastication and facial expression, and willingness to repeat treatment. Counseling for the present protocol should include the 36‐thread configuration, the likelihood of bruising, the potential for asymmetry or dimpling, and the fact that a permanent PP thread may require a removal procedure if it becomes malpositioned or symptomatic.

### 4.6 Safety, Reversibility, and Permanent‐Material Considerations

Bruising occurred in 78/243 patients (32.1%) and resolved within 2 weeks in all recorded cases. Edema occurred in 45/243 (18.5%) and asymmetry in 12/243 (4.9%). These events are important for consent and social downtime counseling even when medically self‐limited. Older patients may also have thinner skin, greater vessel fragility, or medication exposures that affect bruising risk, although the present study did not perform subgroup or risk‐factor analyses. The absence of a major vascular event in this cohort should not be interpreted as proof that such events cannot occur [19, 20].

Risk reduction includes medication review, gentle cannula manipulation, avoidance of repeated passage through the same tract, knowledge of facial vascular and parotid anatomy, and compression when bleeding is suspected. Ultrasound may be useful when the thread pathway is uncertain or when treatment is planned near the parotid gland, parotid duct, facial artery, superficial temporal artery, or other high‐risk structures [29, 30, 31]. Accurate plane control is especially important with permanent threads because a misplaced implant remains present unless it is released or removed.

### 4.7 Persistent Dimpling, Foreign‐Body Complications, and Removal

Dimpling is a mechanism‐based complication that may arise when a thread is excessively superficial, a barb produces focal tethering, tissues are over‐gathered, vectors converge too densely, or the thread changes depth along its course. Early mild dimpling may improve as edema resolves and tissues redistribute, whereas persistent focal depression raises concern for fixed tethering or malposition. Permanent material requires particular caution because spontaneous polymer resorption will not eliminate a persistent mechanical problem.

Two patients required hook‐assisted removal for persistent dimpling (0.8%). One infection and one granuloma were also recorded. Although extrusion and clinically significant palpability were not itemized as separate events in the available cohort summary, they remain recognized concerns with thread lifting and are particularly relevant to permanent implants [4, 20, 30]. A permanent thread may offer continuing support, but its reduced reversibility is a trade‐off: persistent visibility, palpability, asymmetry, infection, granulomatous reaction, extrusion, or malposition may require an intervention rather than observation alone.

The need for removal should not be attributed to material type alone. Depth, barb engagement, vector, tissue thickness, fixation, and facial movement all influence complications. Prevention includes maintaining a consistent safe plane, avoiding abrupt superficialization, reassessing tissue movement during placement, and avoiding excessive local thread density [30, 31]. When a permanent thread is symptomatic or malpositioned, ultrasound‐assisted localization and targeted hook extraction may reduce the extent of surgical exploration [29, 32].

### 4.8 Clinical Implications and Patient Selection

The present experience supports a conservative patient‐selection framework. The clinician should first classify the dominant problem as mild skin laxity, mobile soft‐tissue descent, ligament‐defined folding, jowling, or global heavy‐face ptosis, and then determine whether the expected degree of correction is realistically achievable with threads. Absorbable‐only treatment may be preferable when avoidance of a permanent implant and eventual material resorption are priorities. A combined PDO‐PP approach may be considered when the patient accepts a permanent component and understands that the hypothesized early and later roles of the two materials have not been independently proven in this study.

Vector planning and entry‐point distribution should take precedence over simply increasing thread count. In this protocol, nine entry points per side supported nine PDO and nine PP threads, and redundant trajectories or excessive local density were avoided. Patients should be counseled regarding expected magnitude of improvement, likely bruising, potential asymmetry and dimpling, the possibility of recurrence, and the reduced reversibility of a permanent implant. A complication‐management pathway, including the ability to localize, release, or remove a problematic thread, should be available before treatment.

### 4.9 Photographic Documentation and Outcome Assessment

The longitudinal photographs provide clinically useful context but do not meet the requirements of a standardized objective imaging endpoint. The retrospective archive did not provide consistently standardized frontal, oblique, and lateral views at every time point. Background, camera height, focal length, facial expression, eye position, makeup, hair, neck position, and lighting differed between some image pairs. The figures are therefore retained only as qualitative illustrations of selected patients and should not be used to quantify millimeters of lift, isolate the effect of either thread material, or establish treatment superiority.

Future prospective studies should use fixed camera settings, identical focal length and distance, neutral head position, controlled illumination, hair retraction, and standardized frontal, oblique, and lateral views. Three‐dimensional imaging or other objective methods could quantify vector‐specific displacement and volume redistribution. Blinded evaluators, validated laxity scales, patient‐reported satisfaction, pain, downtime, willingness to repeat treatment, and prespecified adverse‐event surveillance would provide a more rigorous assessment of efficacy and durability.

### 4.10 Generalizability to Other Populations

All participants in this series were Korean patients treated at a single‐center in Seoul. Facial aging patterns, skin characteristics, soft‐tissue distribution, skeletal morphology, and the clinical expression of ptosis may differ across populations. The present findings, therefore, should not be assumed to apply directly to Western Caucasian or other non‐Korean populations, particularly patients with advanced facial laxity. The study is best interpreted as evidence from a defined East Asian clinical population, and external validation in other populations is required before broader generalization.

### 4.11 Limitations

This study has substantial limitations. It was retrospective, single‐center, noncomparative, and based on routine clinical practice. There were no pure‐PDO, pure‐PP, untreated, or surgical comparator groups, so the independent contribution of the thicker PDO component to early mechanical engagement and the permanent PP component to longer‐term support cannot be established. GAIS was physician‐assessed and nonblinded; no objective three‐dimensional or vector‐specific measurement was available, and patient‐reported outcomes were not collected. The Korean‐only cohort further limits generalizability to other populations.

Photography was not standardized and may have been affected by expression, lighting, camera geometry, weight change, hairstyle, makeup, and interval procedures. Follow‐up availability declined from 100% at 6 months to 68.7% at 2 years and 36.6% at 3 years, creating substantial attrition bias. The adverse‐event summary did not provide uniform onset, duration, severity grading, management, or final outcome for edema, asymmetry, infection, and granuloma, and permanent‐material complications can appear beyond the observed follow‐up window. Prior facial procedures were recorded in 18.5% of patients, but their type and timing were not available for stratified analysis.

Despite these limitations, the cohort size is clinically meaningful; all 243 patients had at least 6 months of follow‐up, 167 patients remained evaluable at 2 years, and 89 returned at 3 years. The study therefore adds real‐world information about a relatively large older Korean population treated with a standardized combined thread construct. The findings should be considered hypothesis‐generating and supportive of prospective comparison among pure‐PDO, pure‐PP, combined thread treatment, and appropriate surgical or nonsurgical alternatives rather than definitive evidence of component‐specific benefit, synergy, or superiority.

## 5 Conclusions

Combined PDO‐PP thread lifting was associated with persistent physician‐rated improvement in selected Korean adults aged 60 years or older. Each treatment used 36 threads, all 20 cm long, delivered through nine entry points per side: nine thicker PDO and nine thinner PP threads per hemiface. Mean nonblinded physician‐assessed GAIS declined from 4.2 ± 0.6 at 6 months to 3.5 ± 0.8 at 2 years, while selected qualitative photographs showed residual contour change at 3 years. Bruising occurred in 78 patients (32.1%) and resolved within 2 weeks; edema occurred in 45 (18.5%), asymmetry in 12 (4.9%), infection in 1 (0.4%), granuloma in 1 (0.4%), and two persistent dimples (0.8%) required hook‐assisted thread removal.

The procedural rationale is to use thicker PDO threads for greater initial mechanical tissue engagement and thin permanent PP threads for continuing support, but the absence of single‐material comparator groups prevents attribution of early or late effects to either component and precludes claims of superiority or synergy. Nonstandardized photography, nonblinded outcome assessment, and 36.6% retention at 3 years further limit conclusions regarding durability. In patients with advanced skin excess or cervicofacial ptosis, surgical facelift remains the gold‐standard treatment; thread lifting should be reserved for appropriately selected patients after realistic counseling regarding limited magnitude of correction, recurrence, foreign‐body complications, reduced reversibility of permanent material, and the potential need for thread removal. Prospective controlled studies with standardized imaging, blinded validated scales, patient‐reported outcomes, and long‐term adverse‐event surveillance are needed.

## Author Contributions

All authors have reviewed and approved the article for submission. Conceptualization: Kyu‐Ho Yi, Young Keun Lee. Methodology: Kyu‐Ho Yi, Young Keun Lee, Sung Hun Jung. Investigation: Kyu‐Ho Yi, Young Keun Lee, Sung Hun Jung, Jin‐Su Chung, Guk Hyun Shin, Soo Yeon Park. Data Curation: Kyu‐Ho Yi, Young Keun Lee, Sung Hun Jung, Jin‐Su Chung, Guk Hyun Shin, Soo Yeon Park. Writing – Original Draft Preparation: Kyu‐Ho Yi. Writing – Review and Editing: Kyu‐Ho Yi, Young Keun Lee, Sung Hun Jung, Jin‐Su Chung, Guk Hyun Shin, Soo Yeon Park. Visualization: Soo Yeon Park. Supervision: Kyu‐Ho Yi.

## Funding

The authors have nothing to report.

## Ethics Statement

IRB has been approved by Public Institutional Review Board (P01‐202511‐01‐072).

## Consent

Consent for treatment and retrospective analysis was obtained.

## Conflicts of Interest

The authors declare no conflicts of interest.

## Data Availability

Available upon reasonable request.
